# Moving towards vertically integrated artificial intelligence development

**DOI:** 10.1038/s41746-022-00690-x

**Published:** 2022-09-15

**Authors:** Joe Zhang, Sanjay Budhdeo, Wasswa William, Paul Cerrato, Haris Shuaib, Harpreet Sood, Hutan Ashrafian, John Halamka, James T. Teo

**Affiliations:** 1 Institute of Global Health Innovation, Imperial College London, London, UK; 2 Department of Critical Care, Guy’s and St. Thomas’ NHS Foundation Trust, London, UK; 3 Department of Clinical and Movement Neurosciences, University College London, London, UK; 4 Department of Neurology, National Hospital for Neurology and Neurosurgery, London, UK; 5 Department of Biomedical Sciences and Engineering, Mbarara University of Science and Technology, Mbarara, Uganda; 6 Mayo Clinic Platform, Rochester, USA; 7 Department of Clinical Scientific Computing, Guy’s and St. Thomas’ Hospital NHS Foundation Trust, London, UK; 8 Health Education England, London, UK; 9 London Medical Imaging & AI Centre, Guy’s and St. Thomas’ Hospital NHS Foundation Trust, London, UK; 10 Department of Neurology, King’s College Hospital NHS Foundation Trust, London, UK

**Keywords:** Health care, Medical research

## Abstract

Substantial interest and investment in clinical artificial intelligence (AI) research has not resulted in widespread translation to deployed AI solutions. Current attention has focused on bias and explainability in AI algorithm development, external validity and model generalisability, and lack of equity and representation in existing data. While of great importance, these considerations also reflect a model-centric approach seen in published clinical AI research, which focuses on optimising architecture and performance of an AI model on best available datasets. However, even robustly built models using state-of-the-art algorithms may fail once tested in realistic environments due to unpredictability of real-world conditions, out-of-dataset scenarios, characteristics of deployment infrastructure, and lack of added value to clinical workflows relative to cost and potential clinical risks. In this perspective, we define a vertically integrated approach to AI development that incorporates early, cross-disciplinary, consideration of impact evaluation, data lifecycles, and AI production, and explore its implementation in two contrasting AI development pipelines: a scalable “AI factory” (*Mayo Clinic, Rochester, United States*), and an end-to-end cervical cancer screening platform for resource poor settings (*Paps AI, Mbarara, Uganda*). We provide practical recommendations for implementers, and discuss future challenges and novel approaches (including a decentralised federated architecture being developed in the NHS (*AI4VBH, London, UK*)). Growth in global clinical AI research continues unabated, and introduction of vertically integrated teams and development practices can increase the translational potential of future clinical AI projects.

## Introduction

Multiple indicators over the past five years demonstrate accelerating interest in the application of artificial intelligence (AI) to human health, including exponentially increasing published research since 2016^1^, increasing healthcare provider interest in AI solutions^2,3^, soaring investment into AI startups^4,5^, and year-on-year increases in regulatory approvals^6,7^. In contrast, widespread translation of AI research into implementation remains conspicuously absent, particularly when considering AI for clinical decision-making, diagnosis, or prediction^8^. For example, while high-profile studies demonstrate superiority of AI-assisted cancer detection compared to clinicians^9^, there has been failure to replicate accuracy in larger studies, with limited prospective, real-world validation and poor potential for clinical utility^10^.

Most clinical AI research is conducted on existing, retrospective datasets^11–13^, where focus is on improving algorithm performance for given internal and external datasets, referred to as a ‘model-centric’ approach^14^. Research waste, in the form of algorithms that will never see clinical utilisation, continues to increase^15–17^. Unrepresentative data and model bias contribute to these failings^18,19^, and the push for equitable data accumulation and incremental architectural gains are important for progressing AI as a whole^20^. However, less consideration is given to real-world factors that maintain importance throughout any AI development pathway, including the real-time data lifecycles that support predictions, heterogeneous software and hardware infrastructure that host AI models, and quantification of impact on patients and clinical workflows. Significance of these factors can be seen in failures of previous real-world evaluations of state-of-the-art algorithms, that have been unable to achieve anticipated performance due to infrastructural and data problems^21^, or lack of added value within everyday workflows^22,23^.

In contrast, the use of AI in non-healthcare enterprises has achieved greater success, demonstrating clear return-on-investment^24,25^. Clearly, intricacies and risks inherent to patient data are not comparable to non-healthcare sectors, but lessons can be taken from differing approaches to AI, focussing on value generation, cross-disciplinary collaboration, and a holistic approach to practicalities external to algorithm design^26^.

In this article, we identify practical features of AI development, that have crucial importance for translation, and define their vertical integration within an AI ‘supply chain’. We demonstrate how vertically integrated approaches can work in practice, through the lens of two successful, contrasting, real-world pipelines: a major, scalable, AI platform in a high-resource setting (*Mayo Clinic, Rochester, United States*), and a focused, embedded AI system for cervical cancer screening in a low-resource setting (*Paps AI, Mbarara, Uganda*). We discuss challenges and provide recommendations that can help future AI projects cross the gap from pages of medical journals to patient bedsides.

## Outside of the algorithm

**Fig. 1:**
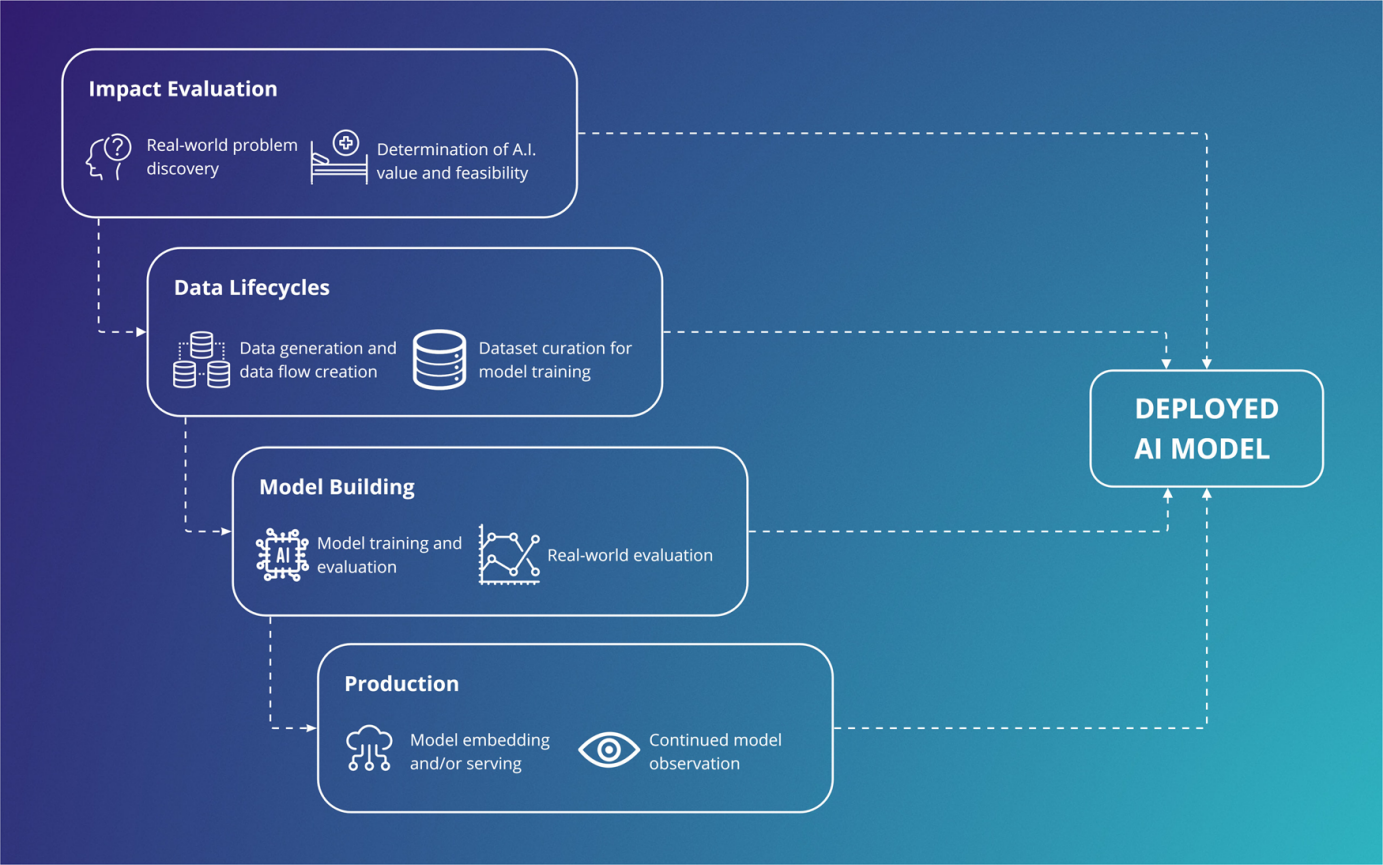
Vertical integration across an artificial intelligence supply chain. All supply chain components are essential for deployment and must work synergistically to support continued AI use. A focus on establishing a supply chain, has benefits over an isolated focus on producing an accurate model.

Increasing attention is being paid to translational aspects of clinical AI^27^. Recent frameworks^28^ and maturity classifications in literature reviews^1,29^ adopt a high-level view of where an algorithm sits in its development roadmap. These supplement checklists for risk of bias and reporting that are internal to algorithm training and evaluation, for prediction^30^ and diagnostic accuracy^31^, which focus on model-building^32^ and generalisability^33^. However, where AI development is intended to lead to clinical deployment, success also depends on practical considerations^34^ outside of model-building (Fig. 1), including impact evaluation, data lifecycles, and production. In the following sections, we describe the contribution of each stage.

### Impact evaluation

AI usage in non-healthcare industries is predicated on creation of ‘value’, measured as tangible return-on-investment. Medical AI is less mature, and most research focuses on accuracy in experimental datasets. Evidence for comparative performance against an existing non-AI gold standard (e.g., AI vs radiologist, or AI + radiologist vs radiologist) is sparser, while evidence of tangible impact on patients or cost-effectiveness is severely lacking. Implementation should aim to generate value for end-users and patients. This could derive from AI-assisted insights with no real-world equivalent (such as associations in complex data), or from augmenting existing skilled clinicians. The latter is particularly relevant for low-income, low-resource environments, or high-resource areas where workstreams are bottlenecked by particular tasks. Impact evaluation therefore includes establishing and estimating clear end-user or patient-centred outcome targets before building a model, as well as planning and monitoring for unintended, post-deployment effects such as over-investigation and over-treatment, or costs from safety-netting high-risk decisions^35^. Involvement of end-users from implementation environments is vital to evaluate real-world impacts, above and beyond a traditional focus on model accuracy^36^.

### Data lifecycles

A healthcare data lifecycle describes generation, curation and aggregation, and maintenance of patient data that is used by consumers (such as clinicians and researchers) and patients themselves^37^. Practical examples can be seen in Learning Healthcare Systems, where analysis is built into daily practice^38^. A lifecycle view emphasises data flows, where data is constantly produced during routine care, and where use and utility of data is often time-constrained. In contrast, model-building is traditionally performed on static datasets that have passed through often unreproducible, proprietary, processing steps. More data, and external data, is not necessarily useful, as additional features may only be available in research settings or through manual collection. While the importance of representative training data is well recognised, other factors can impede successful deployment. These include: (1) differences in how data is acquired and processed, between curated datasets and live implementation environments^39^ (for example – heterogeneous imaging protocols^40^, input and coding of electronic data^41–43^, quality of acquisition device^21^); (2) software and hardware requirements to stream data to a model, which may vary from simple DICOM ingestion to integrating multi-modal data from multiple devices; and (3) a more comprehensive scope of raw data and signals in a live environment, that are not considered by a model, but provide additional insights to a diagnostician (thus reducing relative AI performance).

### AI in production

Production describes the process of bringing an AI model to active deployment. Technical requirements in this stage (including backend/frontend development and product delivery, or “DevOps”), usually call on the expertise of deployment engineers and software developers. As a result, once a promising algorithm has been evaluated by researchers, it will require additional expertise to insert it into the midst of competing software and hardware infrastructure. Successful production enables data ingestion by a model, provides computational power, presents an interface to observe the model working, and returns insights to users. In practice, no two production environments look the same. For example, a model can be embedded into a software application with its own codebase, internal data lifecycle, and user interface. Many imaging algorithms deploy into commonly used radiology workflow software (as seen with segmentation algorithms^6^). Similarly, a model can integrate into physical devices with their own computational power and interface (for example, arrhythmia detecting smartwatches^44,45^). Embedding models takes advantage of discrete workflows and simpler data lifecycles but are ‘locked-in’ to specific uses. A model can instead be served as a module within a larger system, communicating with other modules via interoperable data formats. This approach is scalable, and maintains control over compute, heterogeneous data input/output, flexibility in technology, and resilience in upgrading. However, this comes with high set-up costs and complex development, typically requiring whole organisational buy-in. For models requiring rich data from multimodal sources, this may be the only viable route. Other production considerations include ability to monitor for software bugs and hardware failures, and observe changes in real-world circumstances and data distributions that may cause model performance to deteriorate (‘model drift’) with potential for harm. Models brought to production can encounter difficulties in data interoperability and hardware compatibility, particularly in complex clinical software environments.

### Summary

By the time an AI model, robust to external dataset validation, enters the deployment stage, it may be too late to address challenges related to insufficient clinical impact, inadequate data, and difficulties in production that were not considered in a model-centric approach. These challenges are as important as model-building, with respect to potential for implementation.

## Vertically integrating an AI ‘supply chain’

Vertical integration is a concept from industry that has existed since the 19th century^46^, recognising that supply chain components are co-dependent, and that flow of requirements and information is not unidirectional^47^. Vertical integration synchronises each stage, lowering transaction time and cost to move between them, and renders the entire chain less vulnerable to failure from not anticipating the needs of any individual stage.

We can conceptualise AI development as a vertically integrated supply chain, where model-building is analogous to product construction and testing (Fig. 1). As with traditional product supply chains, this stage does not exist in a vacuum. Rather, operationalisation is entirely dependent on well-functioning components across the chain. Additionally, components must continue to work synergistically to support a deployed model (for example: on-going evaluation of clinical pathway impact, data lifecycles for observation/re-validation, and production environments responsive to safety issues and end-user feedback).

We therefore summarise vertically integrated AI as a holistic approach to AI development, where a focus across the entire supply chain can lead to ready-to-implement models, that are less vulnerable to component failures. In practice, this calls for three actions:

1. To work across all supply chain components in parallel from the planning stage, by aligning to requirements of the final product.
2. To move beyond academically focused groups to cross-disciplinary teams, where end-users, developers and deployment engineers, and implementation experts, play as significant a role as clinician scientists and data scientists.
3. Developing a strategy within research groups, provider organisations, or technology companies, that can facilitate these processes.

**Fig. 2:**
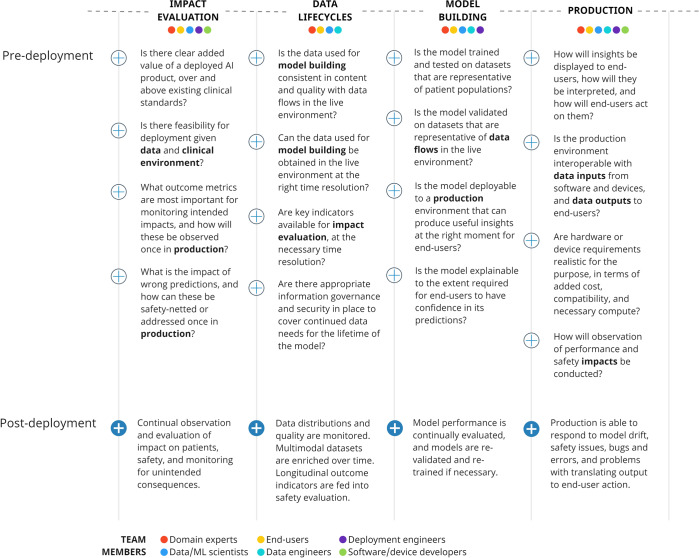
Important considerations across a development supply chain, showing cross-disciplinary involvement across components, that should be addressed early in a vertically integrated approach. With particular relevance to academic circles, broadening of involvement to include users traditionally involved in MLOps (e.g., engineers, developers) can increase translational potential.

For teams looking to create a clinically deployable model, these translate to key considerations in Fig. 2. In the following sections we discuss how this approach is implemented in two AI pipelines with extreme divergence in setting and use-case.

## The Mayo Clinic AI factory – a ‘whole systems’ vertically integrated approach

**Fig. 3:**
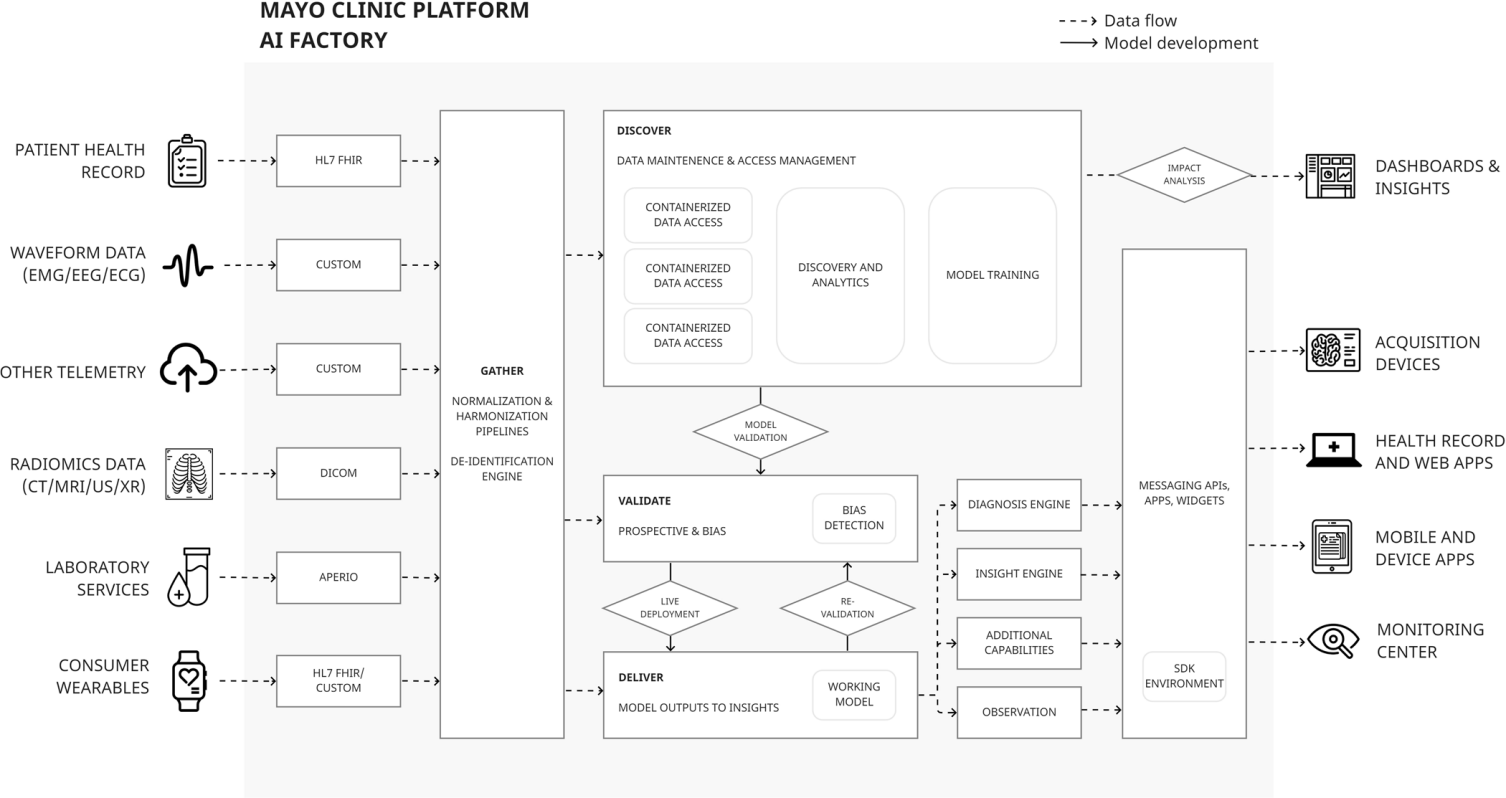
The Mayo Clinic Platform AI factory is a multi-component AI platform that vertically integrates all parts of the AI supply chain into a single infrastructure. This includes components for data curation (“Gather”), data access and analytics (“Discover”), model validation (“Validate”) and an on platform production environment (“Deliver”). This approach, whilst costly, greatly reduces distance from concept to deployment. Cross-disciplinary working is a vital component external to the illustrated architecture.

One strategy for vertical integration is to build an entire organisation-wide infrastructure around AI development. The Mayo Clinic AI factory hosts cross-disciplinary expertise and a development platform, that minimises distance from concept to implementation^48^. Platform architecture is illustrated in Fig. 3. In summary, an interoperable data middle layer (“*Gather”)* utilises multiple Fast Healthcare Interoperability Resource (FHIR) application programming interfaces (APIs) to receive EHR and device/wearables data, with additional APIs integrating imaging and signal (e.g. electrocardiogram) data. Data is hosted in the cloud (*Google Cloud Services, Mountain View, USA*), where reproducible harmonization and quality assurance enables data consistency. Model-builders can access data through the “*Discover*” component, which provides a development environment with compute infrastructure and software tools. Trained models are passed to “*Validate*”, which facilitates silent evaluation on prospective data streams, and automates assessment of model bias by evaluating across population subgroups, and in benchmark datasets for sociodemographic characteristics. “*Validate*” reports performance across a range of scenarios, including calibration and potential for bias in marginalized populations.

Integration of software and computational hardware onto a single platform simplifies production, as implementation can be enabled by switching on a “*Deliver*” component. Model outputs are translated into insights via pre-defined rules, sent to end-users using the existing messaging APIs interfacing with EHR and devices. End-users may be presented with flags, personalized care plans, or access to relevant guidance. Finally, outcome indicators belong to the same data lifecycle and are used to estimate potential impact, or measure intended and unintended post-deployment impacts. The entire platform is supported by a cross-disciplinary team who work with end-users to identify areas of maximal impact, and to vet feasibility with respect to data and production requirements.

A major challenge in implementing a ‘whole systems’ platform is patient privacy. In addition to removing known identifying elements from multimodal data, the platform employs best-on-class de-identification protocols for EHR data (*nference, Cambridge, USA)*^49^. A “data behind glass” approach places authorized sub-tenants in encrypted containers (under Mayo control) but does not allow data to leave containers, preventing merging data with external sources for re-identification.

Launch of the platform in 2020 has resulted in a rich development pipeline^50^. A case-study can be made of ECG-guided screening for asymptomatic left ventricular systolic dysfunction which was taken from conception, through one of the largest randomized control trials of an AI device to date (EAGLE)^51^, and is now undergoing pilot implementation and validation under Food and Drug Administration breakthrough designation. Development is summarised in Table 1, but in short, key contributors to success include: (1) a cross-disciplinary feasibility and planning process; (2) development and deployment supported by interoperable data flows; (3) existing infrastructure that supports regulatory conformity for the whole product lifecycle.

**Table 1:** Pre-deployment and operationalization on Mayo platform of ECG AI-Guided Screening for Low Ejection Fraction (EAGLE).

Supply chain stage	Development pipeline
	Pre-deployment
Impact evaluation	A problem is identified, and a proposed solution is evaluated by a cross-disciplinary team. Prior to deployment, the proposed EAGLE model is judged on (1) potential clinical value, and (2) potential for impactful operationalisation given existing infrastructure and clinical environment. In this case, discovering hidden diagnoses from complex data would provide new diagnostic and screening capabilities that are currently unavailable in the given environment.
Data lifecycles	Availability of suitable datasets and data flows are identified. The team ensures that data flows are available for training, for prospective validation, and for safe monitoring of outcomes. In this case, interoperability between ECG devices and other clinical data within the platform (“Gather”) means that suitable datasets can be curated, accessible in a training environment (“Discover”). Real-time data flows can be easily established for prospective validation, production, and observation. Model output data can be messaged back to end-users at point-of-care.
Model-building	Training a model on data directly curated from real-world pathways Having considered the above, a model trained on the platform can emerge ‘production-ready’. Established data aggregation and quality assurance pipelines on the Mayo platform means accurate and useful labels, allowing EAGLE to be benchmarked in under-represented groups (“Validate”). A well-calibrated model can be taken to prospective validation on live data flows. While in a research container, EAGLE performance can be silently observed against other gold standard diagnostic indicators (such as echocardiography) in the same environment.
Production	Infrastructure that is ready to receive a trained model Positioning of devices and EHR, in parallel to data flows and the model-building environment, means the EAGLE model can be moved directly into a production environment without significant reconfiguration (“Deliver”). Helped by early in-situ end-user involvement, EAGLE outputs will appear directly at a suitable moment on a clinical pathway.
	Operationalization
Impact evaluation + Data lifecycles + Model re-validation + Production	Deployment supported by all components With all components in place, a trained model can be operationalized in a live pathway. Components work symbiotically to support the deployment: 1) Adjacency of analysis and production environment allows users to monitor real-time model outputs. Chosen outcome measures can be observed during a clinical trial^51^. 2) Wider data flows monitored for intended and unintended clinical impacts, contributing to pre- and post-market quality management and compliance with regulatory requirements across the product lifecycle^66^. 3) Containers are created for users to observe data and model output distributions. Early safety signals can trigger model re-validation. Over time, new and manually validated data will enrich the original training dataset. 4) Adjacency of training and production environments, and use of established data flows, means re-validation cycles (and future adaptive AI) are easy to implement. 5) In-situ end-user interactions in development, and once operationalized, allows for direct feedback into usability. Production environment supports responsive updates.

This table describes processes supported by a ready-made vertically integrated infrastructure. The Mayo AI factory maintains close distance between all supply chain components such that ideas can be proposed, evaluated, and operationalized with minimal friction between development stages. For platform architecture, see Fig. 3.

Other use-cases evaluated on the platform include portable ECG assessment^52^, prediction of post-surgical mortality^53^, and real-time monitoring of COVID-19 interventions^54^. More than 200 additional models are in different stages of development maturity^55^, while the platform additionally hosts and accelerates start-ups to market readiness^56^. Vertical integration of data, modelling and validation, production, and clinical impact evaluation into a single platform bridges the gap between algorithms and implementation.

## PapsAI - vertical integration in response to resource scarcity

**Fig. 4:**
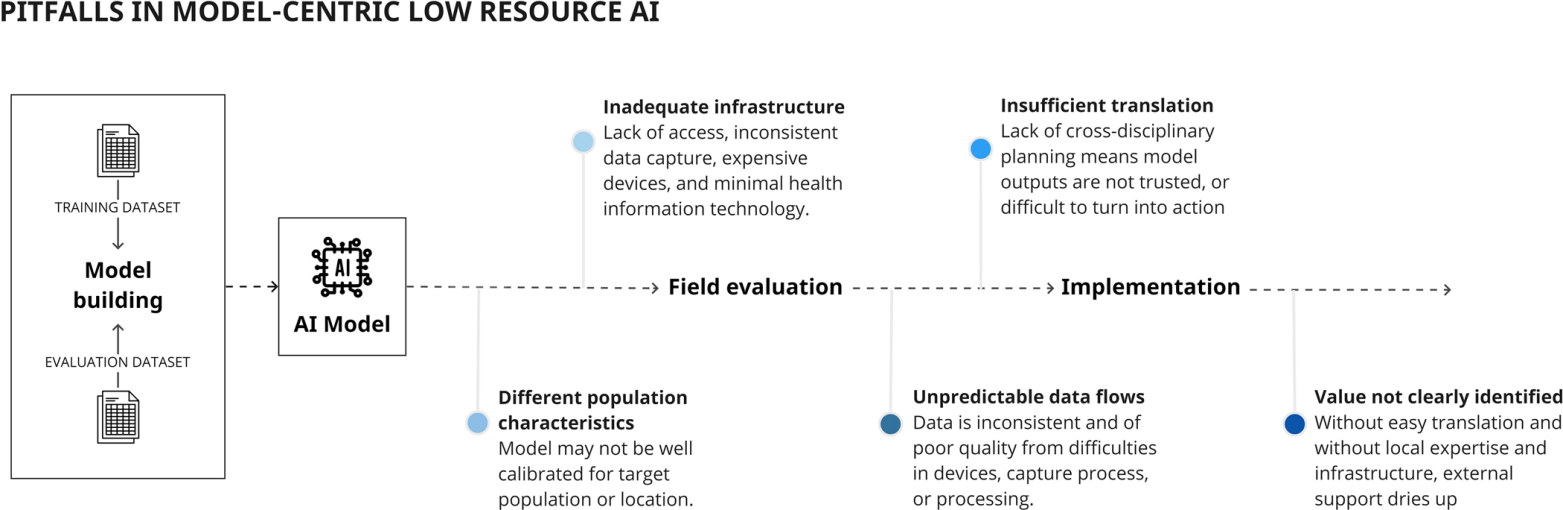
Pitfalls in implementing models specific to lower resource environments. AI models may be trained in high-resource academic labs, and taken to low-resource environments where they fail for the reasons illustrated. A model-centric approach that does not consider real-world supply chain components is unlikely to be successful.

In an opposing, resource-poor scenario, a vertically integrated approach means prioritising infrastructure, and planning for challenging environments where there may not be easy paths to translating model outputs into actions. In such settings, a focus on model-building may produce an algorithm with excellent performance across multiple datasets, but will not address implementation barriers (Fig. 4).

**Fig. 5:**
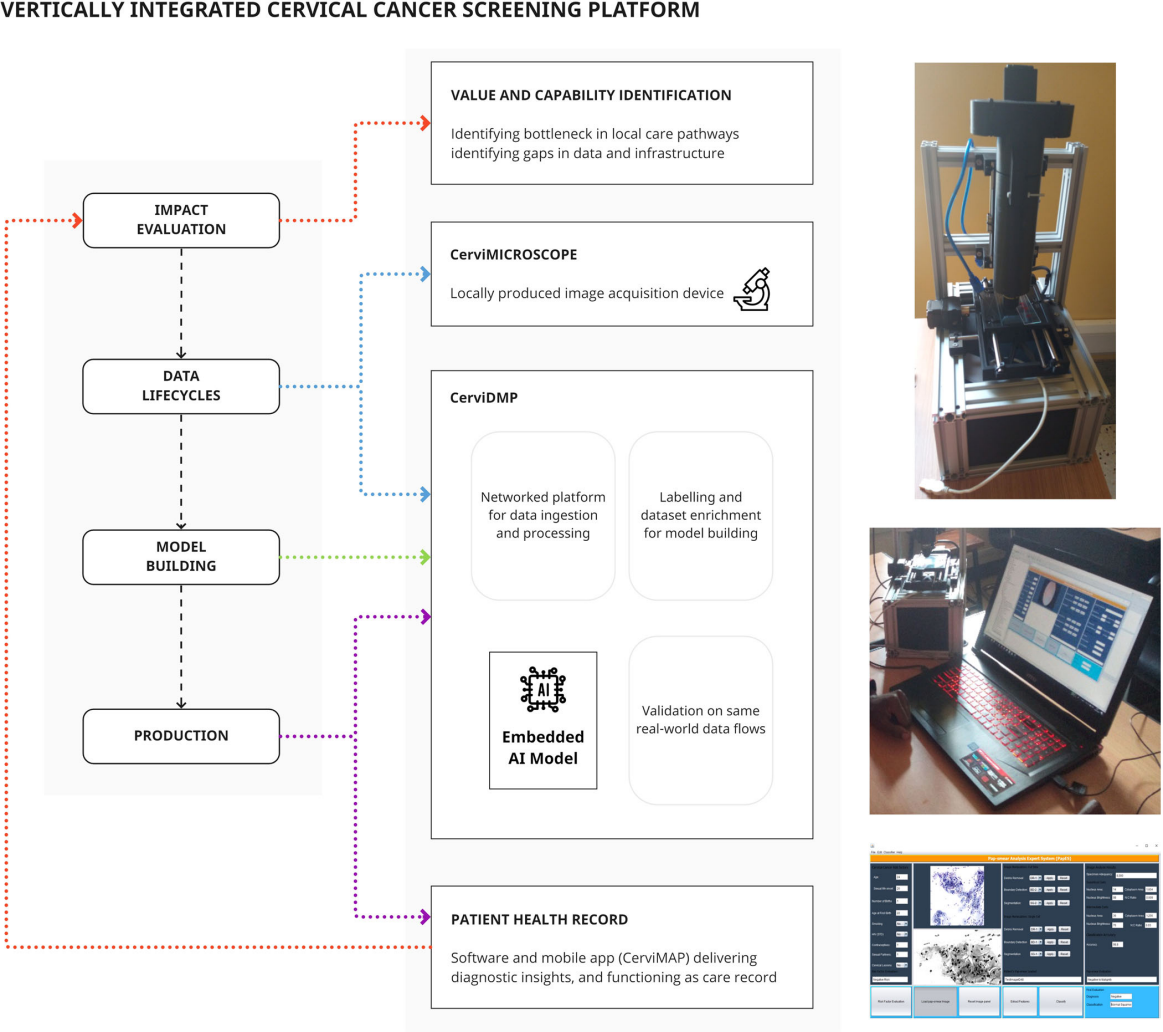
Vertical integration in a cancer screening platform includes parallel development of data and production infrastructure to support model training and implementation. In contrast to Fig. 4, a focus on building supply chain components that support a predictive model will ensure that the model can be operationalized in the real world.

Uganda has high cervical cancer incidence and mortality, with lack of screening resources (included trained cytopathologists) contributing to late diagnosis^57^. Existing algorithms are trained on datasets for high-resource economies with different demographic and data quality characteristics (including cleaner slide preparation) and are designed to integrate with Western cytopathological workflows and expensive devices^58^. Despite a clear use-case for AI, development must contend with a lack of infrastructure. The locally developed approach taken by William et al.^59^ has involved parallel development of hardware and software to support local data lifecycles, with portable training, validation, and production environments, and a health record for outcomes data (Fig. 5), resulting in a ‘ready-to-deploy’ system.

In summary, cytopathological images are acquired through 3D-printed components integrating a microscope slide scanner, networked to an image storage system with labelling/training environment. With parallels to the Mayo Clinic platform (although at a much smaller scale), the same locally applicable data flows are used for model validation. Models are calibrated to local images, include artefact handling, with ability to run on low-end hardware. The production environment sits alongside training software on mobile, low-power devices, and integrates the same data flows. Outputs are added to an electronic record that can be viewed by patients or clinicians and linked to treatment and outcomes. Finally, acquired images can be manually assessed to re-validate the model and enrich the dataset.

There is a paucity of AI research in low to low-middle income countries (LLMIC)^1^, where there is also significant lack of diagnostic resource. Vertical integration promotes local infrastructure – a pre-requisite for representative data and implementation environments. With care, LLMIC AI development can achieve substantially over par return-on-investment, when compared to sums invested into AI for well-functioning clinical pathways in high-resource areas.

## Challenges and solutions in vertical integration

Artificial intelligence has captured the imagination of clinicians and researchers, funders, and commercial investors; but without widespread translation to clinical impact, we risk disillusionment and a collapse in willingness to invest resources.

Vertical integration describes a holistic approach to AI, which engages with all supply components of a planned product at conception, employs teams with cross-disciplinary expertise, and adopts a strategic recognition that model-building in isolation, while often a substantial academic achievement, is not always a practical one.

Practically, approaches will vary across settings. The distance between model-building and other components is a spectrum that differs across data types and clinical environments. The described cases represent two extremes: a large-scale transformation across an organisation, and a planned approach to maximise potential for operationalization in a resource-poor setting. In many other cases, substantial infrastructural changes are unnecessary, as deployment requirements are lower. For example, the dominance of radiomics in development maturity^1^ and devices^8^, may reflect lower implementation requirements from standardized data (DICOM) and pre-existing assisted reporting environments. In addition, the components we describe are not in themselves novel. For companies producing AI software-as-medical-devices, a focus on elements such as software-embedding and value demonstration is necessitated by commercial and regulatory drivers. However, a common feature of the clinical AI research translational gap remains separation of dataset experimentation with ability to operationalise models, as seen in lack of candidates for clinical translation amongst hundreds of COVID-19 models with high reported accuracy^60,61^.

Addressing additional, specific challenges can also help transform current approaches. First, priority for AI funding should be given to proposals with integrated roadmaps to implementation. Statistical methodology could be supplemented by understanding of informatics infrastructure, involvement of deployment experts, and assessment or estimation of longer-term impact. This can be seen in practice, where NHS Transformation in the UK funds projects that fulfil urgent care priorities, and demonstrate feasibility of workflow deployment^62^.

Second, transition to an entirely vertically integrated platform like Mayo Clinic requires whole organisation buy-in. While this can produce ground-breaking results, the required organisational transformation and investment may be unfeasible. Centralisation may be an alternative in regional healthcare networks, accumulating cross-disciplinary expertise from multiple centres, while creating population-level data flows, often through the use of the commercial platform providers^63^. A diametrically opposite approach is decentralisation using federated architectures to manage data environments across multiple organisations, each hosting local cross-disciplinary teams, as being developed by the London Medical Imaging & AI Centre for Value Based Healthcare (AI4BH)^64,65^. While centralisation provides economies of scale for technical specialisation, decentralisation aims to harness synergies through proximity to domain experts and data sources for local requirements.

Finally, medical device regulation and safety monitoring requires reconsideration. Proposed regulation in the USA and UK for a ‘product lifecycle approach’ will consider data flows and production practices (alongside experimental performance metrics)^66,67^. Vertical integration actively supports meeting these regulatory requirements but may also benefit from guidance to address challenging issues such as model and dataset drift, and on-going quality and risk management systems. This dynamic post-translational management stage has been termed ‘MLOps’ by non-healthcare industries^68^.

## Conclusion

Even externally validated and accurate AI models cannot compensate for practical problems that preclude deployment into real-world workflows. Clinical AI development must vertically integrate cross-disciplinary teams and supply chain components that directly support model implementation. This broad approach is adaptable to different settings and can help improve translation of clinical AI research into clinical workflows.

### Reporting summary

Further information on research design is available in the Nature Research Reporting Summary linked to this article.

## Supplementary information

Reporting Summary
